# HLA class I loss and PD-L1 expression in lung cancer: impact on T-cell infiltration and immune escape

**DOI:** 10.18632/oncotarget.23469

**Published:** 2017-12-19

**Authors:** Francisco Perea, Abel Sánchez-Palencia, Mercedes Gómez-Morales, Mónica Bernal, Ángel Concha, Míguela Méndez García, Amanda Rocío González-Ramírez, Martin Kerick, Javier Martin, Federico Garrido, Francisco Ruiz-Cabello, Natalia Aptsiauri

**Affiliations:** ^1^ Servicio de Análisis Clínicos e Inmunología, UGC Laboratorio Clínico, Hospital Universitario Virgen de las Nieves, Granada, Spain; ^2^ Servicio de Cirugía Torácica, Hospital Universitario Virgen de las Nieves, Granada, Spain; ^3^ Departamento de Anatomía Patológica, Universidad de Granada, Granada, Spain; ^4^ Servicio de Anatomía Patológica y Biobanco, Complejo Hospitalario Universitario, La Coruña, Spain; ^5^ Fundación de Investigación Biosanitaria Alejandro Otero, FIBAO, Granada, Spain; ^6^ Instituto de Parasitología y Biomedicina López Neyra, CSIC, Granada, Spain; ^7^ Instituto de Investigación Biosanitaria ibs.Granada, Granada, Spain; ^8^ Departamento de Bioquímica, Biología Molecular e Inmunología III, Universidad de Granada, Granada, Spain

**Keywords:** lung cancer, HLA class I loss, programmed death ligand 1 (PD-L1), tumor infiltrating lymphocytes (TILs)

## Abstract

Immune-checkpoint inhibitors show encouraging results in cancer treatment, but the clinical benefit is limited exclusively to a subset of patients. We analyzed the density and composition of tumor T-cell infiltration in non-small-cell lung carcinoma (NSCLC) in relation to PD-L1 and HLA class I (HLA-I) expression. We found that positive HLA-I expression, independently on PD-L1 status, is the key factor determining the increased density of the immune infiltrate. When both markers were analyzed simultaneously, we identified four phenotypes of HLA-I and PD-L1 co-expression. They demonstrated different patterns of tumor infiltration and clinicopathologic characteristics, including the tumor size and lymphatic spread. All HLA-I+/PD-L1+ tumors had a high degree of intratumoral infiltration with CD8+T-lymphocytes, whereas HLA-I loss was associated with a significantly reduced number of tumor infiltrating T-lymphocytes mostly restrained in the stroma surrounding the tumor nest. HLA-I-negative/PD-L1-positive tumors had bigger size (T) and lower grade of infiltration with CD8+T-cells. It represents a cancer immune escape phenotype that combines two independent mechanisms of immune evasion: loss of HLA-I and upregulation of PD-L1. Using GCH-array analysis of human lung cancer cell lines we found that the loss of heterozygosity (LOH) with complete or partial deletion of HLA-I genes is the principal mechanism of HLA-I alterations. This irreversible defect, which could potentially decrease the clinical efficacy of lung cancer immunotherapy, appears to be underestimated. In conclusion, our results suggest that the analysis of HLA-I is very important for the selection of potential responders to cancer immunotherapy.

## INTRODUCTION

Immunotherapy with antibodies blocking immune checkpoints, including anti-PD-L1, showed durable tumor regression in advanced human cancers [1]. Nevertheless, since only a minority of the patients demonstrates a positive clinical response to check-point inhibitors, the identification of predictive markers and the mechanisms of resistance to immunotherapy become essential. Several studies have proposed that tissue PD-L1 expression, the mutational load, the composition of the inflammatory infiltrate, the presence of Tregs or MDSCs are some of the factors influencing the response to immunotherapy with anti-CTLA-4 and anti-PD-L1 blocking antibodies [2, 3]. There are reasons to believe that the leading cause of the resistance to immunotherapy is the lack of tumor cell immunogenicity, including the loss of tumor expression of HLA class I (HLA-I) antigens, which has been reported in the majority of human malignancies. The expression of HLA-I molecules on tumor cells is an essential factor for the activation of cytotoxic T-lymphocytes. There are only few studies that have analyzed the expression of HLA in the tumor tissue in patients undergoing immunotherapy with “immune checkpoint” inhibitors and only limited data are available on the correlation between the expression of PD-L1 and HLA-I in cancer. It has been known since long time ago that tumors, and particularly non-small cell lung cancer (NSCLC), frequently present alterations in the expression of HLA-I genes, which leads to partial or total absence of these molecules on the surface of tumor cells. This immune escape mechanism is well documented and has been reported and confirmed by us and other groups [4–8]. It has been demonstrated in mouse experimental models and in human cancers that during natural cancer progression tumors gradually lose MHC-I expression as a result of a T-cell mediated immune selection. At early cancer stages MHC-I positive malignant cells are destroyed by cytotoxic T-cells (CTLs), while tumors at more advanced stages become MHC-I negative. This transition to MHC-I negative tumors is also characterized by important changes in tumor microenvironment and tissue re-organization [9, 10]. It has been also shown that the pre-existing CD8+T cells at the invasive tumor margin are closely associated with PD-1/PD-L1 expression and may predict the response to the treatment with antibodies [11]. Hence, density and localization of the CD8+T-cells may substantially affect the clinical efficacy of the anti-PD-1/PD-L1 therapies. Interestingly, a new classification of cancer has been proposed based on the pattern of T-cell infiltration and expression of PD-L1 [12] and four different tumor microenvironment phenotypes have been identified in association with the clinical response to anti-PD-L1 therapy. Recent findings have demonstrated different immune profiles in non-responders, suggesting a potential role of the tumor immune microenvironment in the response to anti-PD-L1 therapy [13].

Recently, Zou and colleagues proposed a definition of two different tumor microenvironment profiles related to the clinical response to anti-PD-1 therapy: ‘inflamed cancer’ characterized by an active response to treatment, and ‘non-inflamed cancer’ with limited or zero response. An ‘inflamed cancer’ responsive to PD-1 pathway blockade could be characterized by several biomarkers, including high levels of PD-L1 expression, Th1-type chemokines, infiltrating T-lymphocytes (TILs), mutations, and low levels of immune suppressive elements [3, 14]. However, we believe that one of the most essential and critical predictive factors of a positive response to immunotherapy is an intact antigen presentation machinery in tumor cells. In this context, in a recent study we found a close association between the HLA-I expression and an inflammatory pattern in tumor tissues [15]. We characterized two types/stages of tumor tissue organization: so-called “permissive” type, with T-cells infiltrating tumor mass in a direct contact with HLA-I positive cancer cells; and a “non-permissive” structure with TIL-free HLA-I negative tumor encapsulated by stroma. The latter one is an example of tumor escape phenotype with high probability of resistance to immunotherapy, since it is negative for HLA-I expression and has a dense mesh of stromal tissue surrounding tumor nest, which restrains CD8+ T-lymphocytes and other immune cells [15].

Here we examined the density of tumor infiltration, its composition and association with the expression of PD-L1 and HLA-I in both freshly collected cryopreserved and paraffin-embedded tissues from patients with NSCLCs. We analyzed the correlation between the obtained data and patients´ clinicopathologic characteristics in order to identify potential predictive biomarkers of response to cancer immunotherapy.

## RESULTS

### Immunohistochemical analysis of PD-L1 and HLA-I expression in human NSCLCs samples

**Figure 1:**
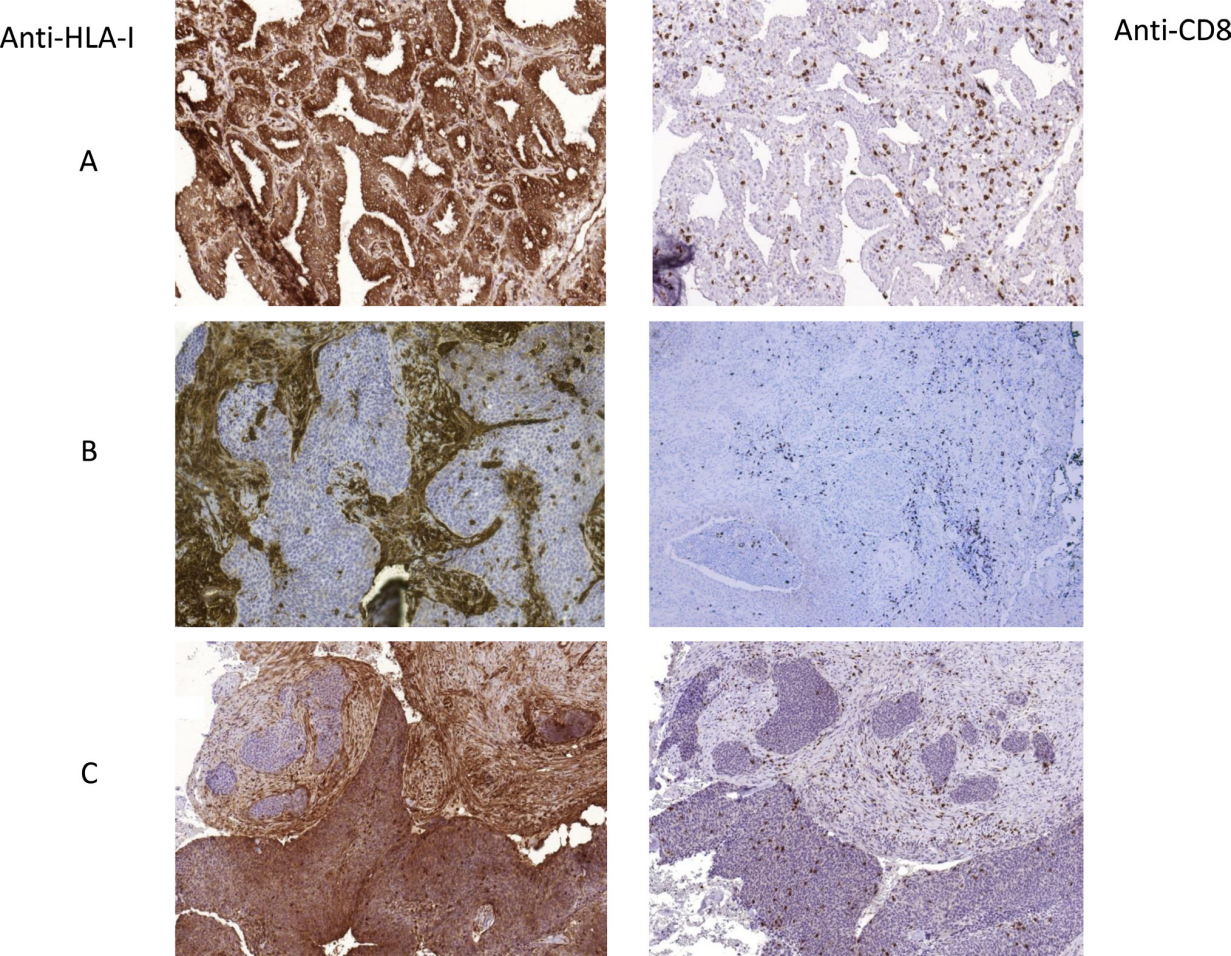
Representative images of tumor HLA-I expression and CD8+ T-cell infiltration patterns (**A**) HLA-I positive and highly infiltrated cryopreserved tumor, **(B)** HLA-I negative tumor with mostly peritumoral stromal infiltration pattern, **(C)** tumor with heterogeneous HLA-I expression and heterogeneous pattern of infiltration. Intratumoral T-cells in HLA-I positive zone and stromal/peritumoral T-cell localization in HLA-negative areas. In B and C the samples were paraffin-embedded. All Images are at 10× magnification.

The expression of HLA-I antigens was evaluated in 68 lung cancer tissue samples by Immunohistochemical staining using antibodies against monomorphic determinants of HLA-ABC complex, locus-specific antibodies and anti-β2-microglobulin (B2M) antibodies. The most frequent alteration detected in cryopreserved sections was a total loss or downregulation of HLA-ABC molecules, which was detected in 30 out of 68 cases (44%), although we also observed partial losses of HLA-A and HLA-B loci. No significant association was found between HLA-I expression and clinicopathologic characteristics of the patients (Table 1). Figure 1 depicts representative images of different HLA-I immunolabeling patterns in the studied tumor samples.

**Table 1:** Clinical features of lung cancer patients and tumor HLA class I expression

Clinical Features	HLA-I expression		*p*-value
	Positive or heterogeneous *n* = 38 (56%)	Negative *n* = 30 (44%)	
Age			
Mean: 67 (45–82)			
Sex			
Male: 53 (78%)	26 (68%)	27 (93%)	0.014
Female: 15 (22%)	12 (32%)	3 (7%)	
Smoking History			
Somker: 59 (88%)	34 (90%)	25 (86%)	0.719
No smoker: 9 (12%)	4 (10%)	5 (14%)	
Primary Tumor			
T1:19 (30%)	11 (31%)	8 (28%)	0.790
T2: 36 (56%)	20 (57%)	16 (55%)	
T3 and T4: 9 (14%)	4 (12%)	5 (17%)	
Nodal status			
N0: 49 (78%)	28 (80%)	21 (75%)	0.635
N1 and N2: 14 (22%)	7 (20%)	7 (25%)	
Tumor stage			
I: 34 (54%)	20 (57%)	14 (50%)	0.572
II and III: 29 (46%)	15 (43%)	14 (50%)	
Grade			
Well/moderate: 37 (57%)	23 (64%)	14 (48%)	0.206
Poor: 28 (43%)	13 (36%)	15 (52%)	
Histology			
Squamous cell carcinoma: 32 (49%)	17 (47%)	15 (52%)	0.432
Adenocarcinoma: 31 (48%)	17 (47%)	14 (48%)	
Large cell carcinoma: 2 (3%)	2 (6%)	0 (0%)	

**Figure 2:**
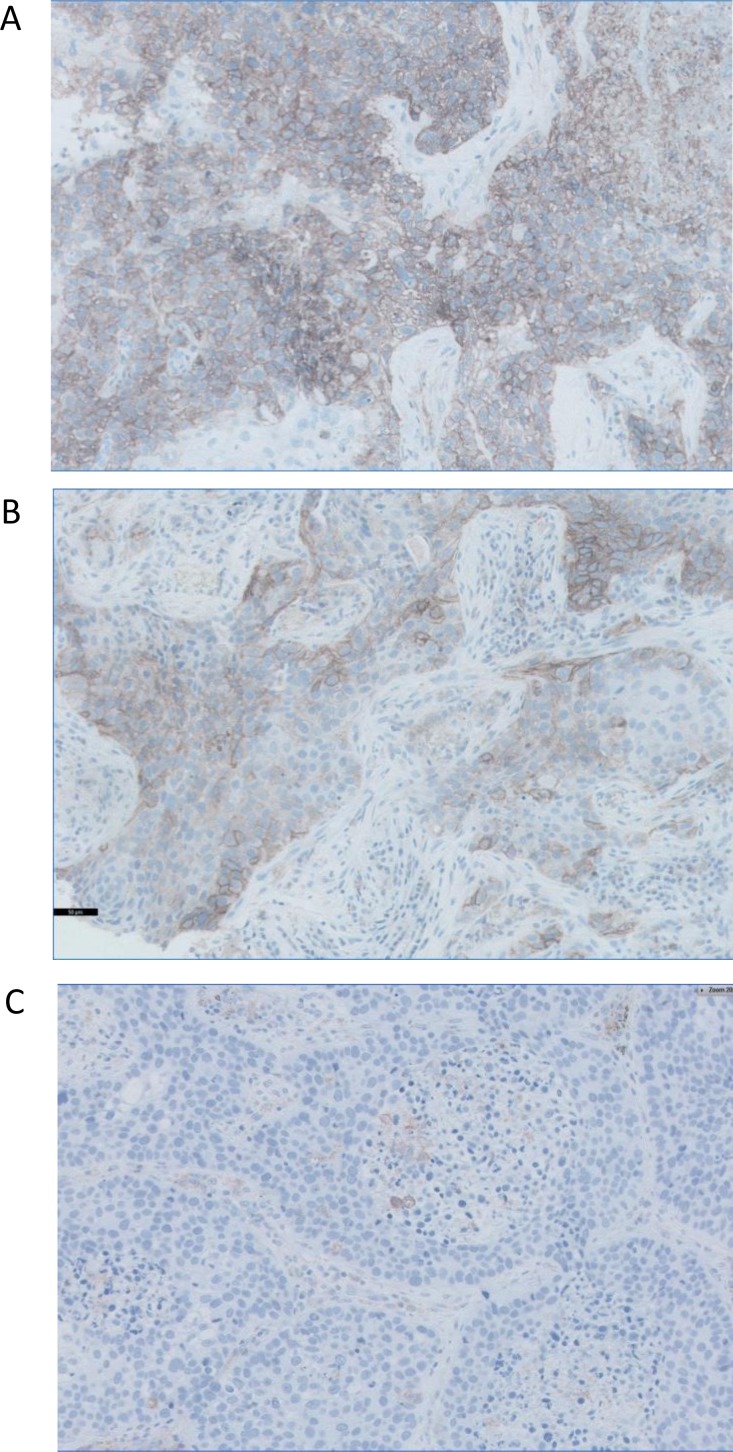
Representative images of three paraffin-embedded tumors with different patterns of PD-L1 expression (**A**) homogeneously positive, (**B**) heterogeneous pattern with PD-L1-positive and -negative cell, and (**C**) negative PD-L1 expression. Immunohistochemistry kit PD-L1 22C3 pharmDx was used to detect the expression of PD-L1, which was defined as positive when tumor cells showed a membranous staining of any intensity. All Images are at 40x magnification.

The expression of PD-L1 by immunohistochemistry was evaluated in 52 tumors (Table 2) and three different patterns of expression were observed: negative for PD-L1 (31 samples out of 52), positive PD-L1 expression and heterogeneous PD-L1 expression (21 tumors altogether). In PD-L1 negative tumor samples some stromal cells, morphologically similar to macrophages, were labeled positively for PD-L1. In tumors with heterogeneous PD-L1 expression some tumor cells and macrophages were labeled positively. In PD-L1 positive tumors the majority of tumor cells were positive with various intensity of staining. For statistical analysis we classified all the tumors in two groups: PD-L1-negative tumors and positive/heterogeneous tumors. Representative staining patterns of PD-L1 expression in the studied tumors are shown in Figure 2. Correlation between the PD-L1 expression and clinical characteristics of the patients are summarized in Table 2. We did not see any significant correlation between PD-L1 expression and most of the clinical parameters. However, we found an association between positive PD-L1 expression and tumor stage. The majority of PD-L1positive/heterogeneous tumors were at more advanced stages II+III as compared to PD-L1-negative tumors (Table 2).

**Table 2:** Clinical features of lung cancer patients and tumor PD-L1 expression

Clinical Features	PD-L1 expression		*p*-value
	Positive or heterogeneous *n* = 21 (40%)	Negative *n* = 31 (60%)	
Age			
Mean: 67 (45–82)			
Sex			
Male: 39 (75%)	18 (86%)	21 (68%)	0.142
Female: 13 (25%)	3 (4%)	10 (32%)	
Smoking History			
Somker: 46 (88%)	20 (95%)	26 (84%)	0.382
No smoker: 6 (12%)	1 (5%)	5 (16%)	
Primary Tumor Grade			
T1: 12 (25%)	4 (22%)	8 (27%)	0.494
T2: 26 (55%)	9 (50%)	17 (59%)	
T3 and T4: 9 (20%)	5 (28%)	4 (14%)	
Nodal status			
N0: 33 (72%)	10 (59%)	23 (79%)	0.181
N1 and N2: 13 (28%)	7 (41%)	6 (21%)	
Tumor stage			
I: 20 (44%)	4(24%)	16 (55%)	0.037
II and III: 26 (56%)	13 (76%)	13 (45%)	
Grade			
Well/moderate: 26 (53%)	10 (53%)	16 (53%)	0.962
Poor: 23 (47%)	9 (47%)	14 (47%)	
Histology			
Squamous cell carcinoma: 23 (46%)	11 (57%)	12 (39%)	0.345
Adenocarcinoma: 25 (50%)	7 (38%)	18 (58%)	
Large cell carcinoma: 2 (4%)	1 (5%)	1 (3%)	

We did not find any significant correlation between HLA-I and PD-L1 expression. There were both HLA-I positive/PD-L1 negative and HLA-I negative/PD-L1 positive cases. Although there was no direct association between tumor stage/grade and HLA-I or PD-L1 expression analyzed separately, we saw interesting results when we combined HLA-I and PD-L1 expression and classified tumors into the following four groups: 1) HLA-I+/PD-L1+, 2) HLA-I+/PD-L1–, 3) HLA-I-/PD-L1+ and 4) HLA-I-/PD-L1–. We found an association between HLA-I/PD-L1 expression and tumor grade (Table 3) and were able to demonstrate what impact has HLA-I expression in PD-L1-positive tumors, as well as the value of PD-L1 expression in HLA-I negative tumors. Thus, HLA-I expression appeared to be an important factor associated with primary tumor size (T) in PD-L1-positive tumors: the majority of HLA-I–/PD-L1+ tumors had larger tumor size (T3+T4), as compared to HLA-I+/PD-L1+ tumors (*p* < 0.023) and HLA-I–/PD-L1- tumors (*p* < 0.008) (Table 3).

**Table 3:** Correlation between primary tumor (T), HLA-I/PD-L1 expression and lymphocyte infiltration pattern

HLA-I Expression	Positive		Negative			
PD-L1 Expression	^a^Positive	Negative	^a^Positive	Negative		*p*-value
Primary Tumor (T)						
T1:12	1 (10%)	6 (43%)	3 (38%)	2 (13%)		^b^0.023
T2: 26	8 (80%)	5 (36%)	1 (12%)	12 (80%)		^c^0.008
T3 and T4: 9	1 (10%)	3 (21%)	4 (50%)	1 (7%)		
Infiltration pattern						
TILs: 34	13 (100%)	11 (73%)	3 (37%)		7 (44%)	^d^0.003
Stromal : 18	0 (0%)	4 (27%)	5 (63%)		9 (56%)	^e^0.001

^a^PD-L1 positive tumor group includes tumors with both heterogeneous and homogeneous expression patterns.

^b^*p*-value for the difference between HLA-I–/PD-L1+ with HLA-I+/PD-L1+ tumor phenotypes and T2 and T3+T4 grades.

^c^*p*-value for the difference between HLA-I–/PD-L1+ with HLA-I–/PD-L1– tumor phenotypes and T2 and T3+T4 grades.

^d^*p*-value for the difference between HLA-I+/PD-L1+ with HLA-I–/PD-L1+ tumor phenotypes.

^e^*p*-value for the difference between HLA-I+/PD-L1+ with HLA-I–/PD-L1– tumor phenotypes.

### HLA-I/PD-L1 tumor expression and immune infiltration

**Figure 3:**
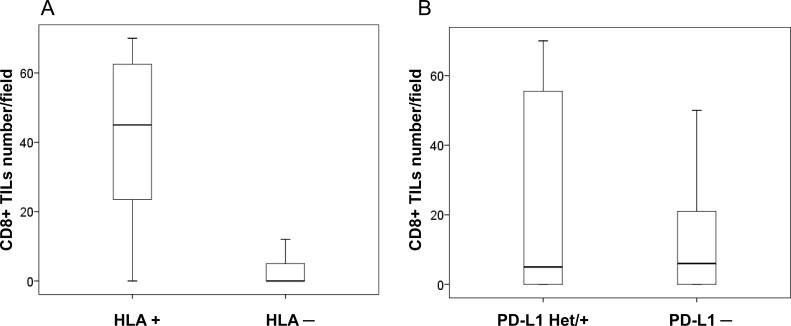
Immune infiltration and HLA-I and PD-L1 expression in cryopreserved tumor samples (**A**) HLA-I positive tumors with higher degree of CD8+T-cell infiltration according to cell counts (*p* < 0.05); (**B**) no statistically significant difference in CD8+ infiltration between PD-L1 positive/heterogeneous and PD-L1 negative tumors (*p* = 0.250). U Mann Whitney test was used to evaluate statistical differences.

We analyzed inflammatory infiltrate in stained NSCLC samples, including the distribution and quantification. Figure 1 depicts representative images of tumor infiltration with CD8+ T-cells. None of the analyzed cell populations (CD45+, CD45RO+, CD3+ and CD8+) showed any association with the clinicopathologic characteristics of the tumors/patients. However, analysis of the association between HLA-I expression and tumor infiltration revealed that the mean rank of CD8+ cell numbers was significantly higher in HLA-I positive tumors than in HLA-I negative ones, (*p* < 0.0001) (Figure 3A).

**Figure 4:**
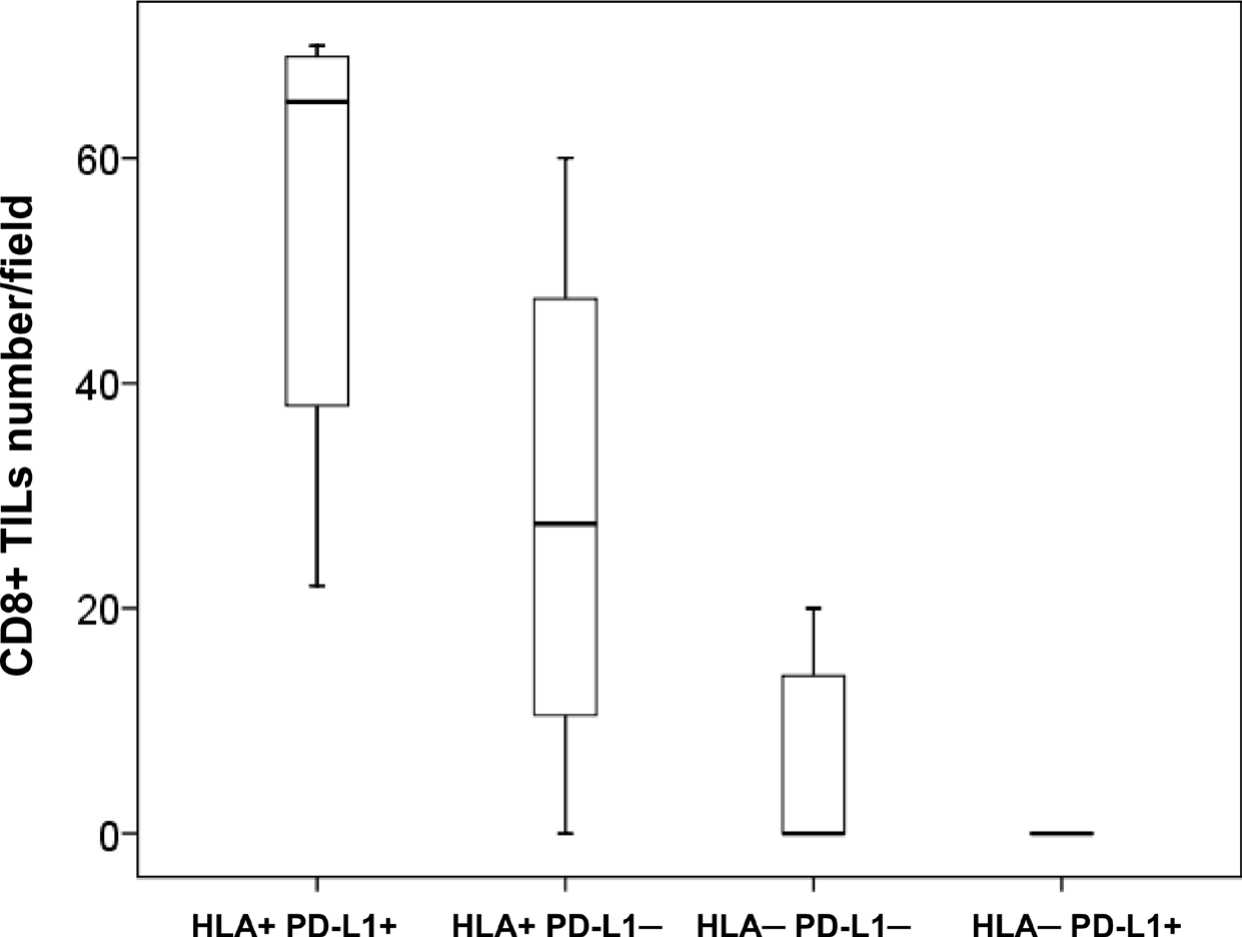
Combined HLA-I/PD-L1 expression phenotypes and CD8+ T-cell infiltration in cryopreserved NSCLC tumors HLA-I+ tumors (with or without PD-L1 expression) showed a greater infiltration with CD8+ cells than HLA-I–/PD-L1+ tumors (measured as number of cells per field at 40x magnification) (*p* = 0.000036) . Kruskal- Wallis test was used to evaluate statistical differences between the groups.

The analysis of the association between PD-L1 expression and infiltration did not reveal statistically significant correlations. We found a greater infiltration in PD-L1 positive/heterogeneous than PD-L1 negative tumors, but the differences were not statically significant (*p* = 0.250, Figure 3B). Importantly, when we combined the expression of HLA-I and PD-L1 we found that the phenotypes with the highest degree of tumor infiltration with CD8+ T-cells were HLA+/PDL+ and HLA+/PDL1–, while HLA–/PDL+ tumors almost did not have any intratumoral lymphocytes (Figure 4).

In a previous study we were able to distinguish different patterns of infiltration and localization of different immune cell subpopulations in relation to tumor nest and stroma: 1) pattern of intratumoral infiltration with T-cells and macrophages and only few immune cells in the stroma; 2) stromal pattern with lymphocytes/macrophages residing only outside the tumor nest in the surrounding stroma [21]. We also observed a significant correlation between tumor HLA-I expression, the infiltration pattern and tumor/stroma tissue organization. Tumors with HLA-I expression demonstrated a “permissive” tissue architecture when tumor cells have a direct contact with infiltrating CD8+ T-cells and are not surrounded by highly organized stromal margin. On the contrary, tumors with lost or significantly reduced HLA-I expression showed predominantly peritumoral localization of activated immune cells with tumor nest encapsulated by well-formed stromal tissue.

Here we investigated the association of these two types of tumor tissue patterns with the expression of PD-L1. Based on the obtained results we classified tumors into following groups based on the expression of HLA-I and PD-L1: 1) HLA-I+/PD-L1+, 2) HLA-I+/PD-L1–, 3) HLA-I–/PD-L1+ and 4) HLA-I–/PD-L1-. We discovered that the infiltration pattern depends on the HLA-I phenotype more than on the expression of PD-L1. Interestingly, in tumors with heterogeneous HLA-I expression we were also able to see such an HLA-I-dependent distribution of CD8+ T-lymphocytes (Figure 1), where TILs can be seen only in HLA-I-positive tumor nests, while in HLA-I negative areas tumor-infiltrating cells are restricted only to the stromal area in the tumor margin. Among all the observed HLA-I/PD-L1 combinations (Table 3), HLA-I negative tumors demonstrated higher incidence of a stromal infiltration along the tumor border without TILs, while all double positive tumors had intratumoral infiltration. Thus, 100% of the HLA-I+/ PD-L1+ tumors had TILs, while among tumors with loss of HLA-I and positive expression of PD-L1 (HLA-I–/PD-L1+) the percentage of tumors with TILs was reduced to 37% (*p* < 0.003, Table 3). Similarly, a comparative analysis of HLA-I+/PD-L1+ and HLA-I–/PD-L1– tumors demonstrated that double negative tumors have lower incidence of intratumoral lymphocyte infiltration compared to double positive ones (*p* < 0.001) (Table 3).

### Analysis of HLA-I and PD-L1 expression in human lung cancer cell lines

As shown in Supplementary Table 1, all the studied cell lines demonstrated positive but variable levels of expression of HLA-I y PD-L1 as measured by flow cytometry. HLA-I expression was positively correlated with qRT-PCR levels of expression of B2M and NLRC5, a transcriptional factor regulating HLA-I expression (Supplementary Figure 1). However, we did not see a correlation with mRNA expression levels of HLA-I heavy chain and components of the antigen presentation machinery (APM), including TAP1/2, LMP2/7, tapasin, and calreticulin (data not shown). Notably, loss of HLA-I haplotype detected in some cell lines (Supplementary Table 2) did not result in reduced cell surface expression of HLA-I. Thus, SK-LU-1 y CALU-6 cell lines have second and third highest MFI numbers despite the loss of one HLA-I heavy chain gene copy due to the haplotype loss. IFN-gamma treatment increased HLA-I expression in all studied cell lines except for SK-LU-1 cells. PD-L1 expression both in baseline conditions and after incubation with IFN-gamma also varied between the cell lines (Supplementary Table 1).

### Loss of heterozygosity (LOH) in HLA-I region of chromosome 6 in human lung cancer cell lines

**Figure 5:**
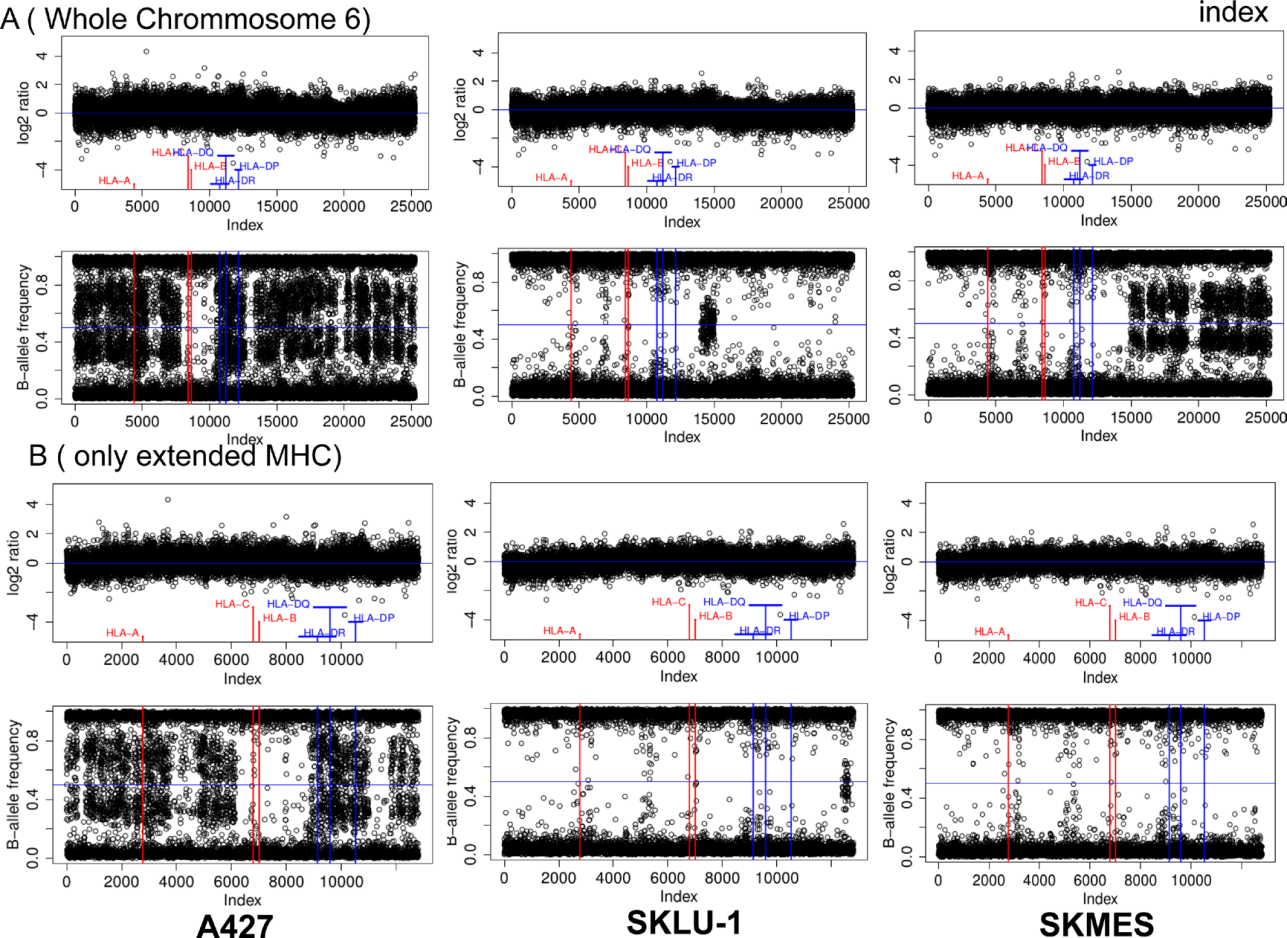
HLA-I gene deletion in human lung cancer cell lines Joined copy number (CN) and loss of heterozygosity (LOH) analysis of (**A**) chromosome 6 and as a zoom (**B**) the extended MHC region of three cell lines (A427, SKLU-1, SKMES). The upper part of each subfigure depicts the CN analysis in the form of Log2-ratios while the lower part shows the corresponding LOH analysis in the form of B-allele frequencies. Log2 ratios were calculated by comparing the samples fluorescence values (Illuminas Genome Studio: “R”) to a standard obtained as the median of 1632 unrelated samples. All values have been plotted equidistant ordered by chromosomal position to facilitate the detection of LOH and CN aberrations.

The results of HLA genotyping in the studied cell lines showed homozygosity for the HLA-A/B/C and HLA-DR/DP/DQ genes in 3 out of 6 cell lines and homozygosity for HLA-B/C in cell line A-427 (Supplementary Table 2). Since we did not have autologous normal DNA corresponding to the studied cell lines we investigated the possibility of loss of heterozygosity (LOH) in the chromosome 6 using Infinium Global Screening Array-24 (Figure 5). A joined analysis of copy number (CN) and beta-allele frequencies of chromosome 6 using a genotyping array demonstrated extended LOH in SK-LU-1 and CALU-6 (not shown) on both arms of chromosome 6. LOH was also observed in A427 cells, but restricted to the HLA-B and HLA-C region. SK-MES cells showed intermediate results, with extensive LOH only on the short arm of chromosome 6. LOH analyzed together with the CN data uncovered a complex pattern. A427 cells showed no CN changes in LOH regions, while SKLU-1 showed deletions in LOH regions of HLA Class I and II genes. SK-MES have the most CN aberrations with deletions in all classical HLA alleles (A/B/C/DR/DP/DQ). Taken together, obtained results of HLA genotyping and gene arrays suggest a pattern of hemizygosity indicating the deletion of all or part of the HLA region. These results are comparable with the data previously obtained from microdissected tumor nests of HLA-I negative tumors [21].

## DISCUSSION

Reduced expression of tumor HLA-I is an important immune escape mechanism from cytotoxic T-cells (CTL) described in various types of cancer, which frequently is associated with poor prognosis and resistance to immunotherapy [4–8].

Antibodies blocking co-inhibitory immune checkpoint molecules PD-1 and PD-L1 improve the ability of the immune system to attack tumors cells, including lung cancer. However, many patients either do not show any clinical response or do not develop durable response [16]. Hence, identification of the factors that can predict the efficacy of these treatments is yet to be defined. Tumor expression of PD-L1 has been suggested as one of such predictive biomarkers along with tumor immunogenicity and tumor microenvironment with T-cell infiltration, which had been already recognized as positive prognostic indicators in a variety of cancers [17–22]. However, the efficacy of “immune checkpoint” blocking antibodies targeting PD-1/PD-L1 axis depends on tumor expression of HLA-I molecules and the limited clinical efficacy of immunotherapy could be partially explained by the tumor HLA-I loss [4–8, 17] . Only very limited data have been reported to date on the correlation between the expression of PD-L1 and HLA-I in different human tumors.

In this study, we aimed to analyze NSCLC for the expression of tumor PD-L1 and HLA-I in association with the pattern of immune cell infiltration to identify prognostic and predictive markers of cancer progression and response to immunotherapy. Based on the obtained results we grouped studied tumors in four distinct phenotypes of HLA-I and PD-L1 expression, which potentially could have a different association with the response to cancer immunotherapy with antibodies blocking the PD-1/PD-L1 axis. These tumor immunophenotypes showed considerably different patterns of tumor inflammatory infiltration (both the type and the density of the infiltrating immune cells) associated with certain clinicopathologic characteristics, including primary tumor size (T).

Based on the existing knowledge of the mechanisms of cancer immune escape and modulation of the immune response, loss of tumor HLA-I together with positive expression of PD-L1 represent two routes of natural adaptive tumor immune evasion mechanisms and might contribute to the resistance to anti-tumor immunity [17, 23–27]. The consequence of any of these immune evasion mechanisms can be fundamentally different in the context of immunotherapy with immune checkpoint inhibitors. In this study, tumor expression of PD-L1, of HLA-I and the immune infiltrate subsets (when studied separately) did not show any correlation with the clinicopathologic parameters. However, according to our results, tumor expression of HLA-I (but not of PD-L1) determines the pattern (Figure 1) and the density of CD8+ infiltration (Figure 3A). Similarly, we observed that CD45RO memory T-cells (but not CD45, CD3 and T-regulatory Foxp3+ cells) prevail in HLA-I positive as compared to HLA-I negative tumors (*p* < 0.007) (data not shown). When we analyzed the HLA-I and PD-L1 expression together (as a joint tumor immunogenicity characteristic), we found a significant correlation with the clinical characteristics of the patients. Tumors with HLA-I–/PD-L1+ phenotype showed greater primary tumor extension (T) (Table 3), lymphatic spread and less tumor-infiltrating CD8+T-cells (Figure 4) as compared to HLA-I+/PD-L1+ tumors (*p* < 0.023). In addition, HLA-I–/PD-L1– tumors had smaller size (T) than HLA-I–/PD-L1+ tumors (*p* < 0.008, Table 3), while the majority of HLA-I+/PD-L1– tumors (64%) were at the early stage I of cancer development (data not shown). These data taken together indicate that HLA-I+/PD-L1– expression pattern is favorable for tumor rejection, while HLA-I–/PD-L1+ phenotype is more aggressive from the immunological point of view and can be responsible for the resistance to immune checkpoint therapy in patients with high tumor PD-L1 expression [28].

Remarkably, we also found differences between these immunophenotypes in relation to the localization of CD8+ T-cell infiltrate. HLA-I+/PD-L1+ tumors more frequently show a pattern of intratumoral infiltration, which allows a direct contact with tumor cells (Figure 1). On the contrary, the localization of T-cells in HLA-I–/PD-L1+ tumors is mostly peritumoral/stromal (*p* < 0.003). These two immunophenotypes (HLA-I+/PD-L1+ versus HLA-I–/PD-L1+) could potentially be associated with the response versus resistance to immunotherapy with tumor HLA-I expression being a pivotal factor and a driving force of tumor rejection in response to immunotherapy.

Teng and co-authors have recently proposed a classification of tumors based on the pattern of tumor PD-L1 expression and infiltration with T-cells [18]. In general, assessment of PD-L1 and the tumor immune contexture as potential predictive markers of the response to checkpoint blockade have some controversies and limitations. It could be attributed to the limitations of the immunohistochemical analysis when only T-cell infiltration is evaluated, and which is not enough to define the complexity of the tumor microenvironment [29–32]. This complexity of the immunoscore evaluation increases due to the fact that infiltration patterns are often heterogeneous even within the same tumor specimen (Figure 1). In addition, the difference in the response may be also associated with the requirement of an increased density of CD8+ T cells in close proximity to PD-1/PD-L1 expressing cells at the invasive tumor margin and inside the tumors [11, 33]. Furthermore, the optimal level of tumor PD-L1 expression necessary for an adequate anti-tumor immune response remains to be established.

We strongly believe that tumor HLA-I expression should be included into the evaluation of patients for therapy selection for several reasons. First of all, the recognition of tumor peptides presented by HLA-I to CD8+ T- lymphocytes is the most critical stage during their activation. Second, HLA-I expression is frequently altered in tumors of different histological types [4, 27, 34]. Third, the emergence of HLA-I negative tumor variants by B2M mutations and defects in genes that regulate the IFN-gamma signaling, occurs during the natural history of cancer [26, 35] or after immunotherapy as a result of immune selection [19, 36, 37]. Finally, natural resistance to cytotoxicity may be irreversible if is caused by mutations in B2M gene or LOH in chromosome 6 harboring HLA-I heavy chain genes.

We have data demonstrating that HLA-I loss caused by B2M mutations is not very common in NSCLC [15] in contrast to what has been reported in colorectal cancer and melanoma [34]. However, LOH seems to have relevance as a mechanism of HLA-I loss in NSCLC.

We have previously observed a total HLA-I loss associated with two simultaneous defects, LOH in chromosome 6 and a downregulation of HLA-I and APM genes [21]. We have reasons to believe that the frequency of LOH in cancer, especially in NSCLCs, is substantially underestimated. In this context, the data obtained in this study from flow cytometry of six lung cancer cell lines demonstrated that HLA-I positive cells have notable variations in the levels of cell surface expression of HLA-I complex (Supplementary Table 1). However, neither flow cytometry nor immunohistochemical analysis using monomorphic monoclonal antibodies alone can reveal the complexity of all possible alterations in the MHC genes in NSCLCs. In fact, in our study 4 out of 6 cell lines showed total or partial HLA haplotype loss (Figure 5). Furthermore, SK-MES harbored two different alterations: complete LOH of HLA class I and II genes and loss of response to IFN-gamma (Figure 5 and Supplementary Table 1).

These data emphasize the complexity of predicting patients´ responses to immunotherapy if all the indicated parameters are not taken into account. Although PD-L1 expression have been associated with the cancer immune response in some patients submitted to immunotherapy, we believe that tumor HLA-I expression is the critical factor driving the positive response and determining the efficacy of the therapy. In fact, we believe that HLA loss can explain the delayed relapses observed in some patients after initial tumor regression and despite continuous therapy.

## MATERIALS AND METHODS

### Clinical and histopathological characteristics of tumor samples

68 NSCLC (non-small cell lung cancer) patients were included in this study. Informed consent approved by the Ethics Committee of our institution was signed by all patients included in this study. Before the study, all medical records and tumor sections were reviewed by an oncologist and a pathologist. Tumor samples were obtained from the Biobank of the Virgen de las Nieves University Hospital (Granada, Spain) and Complejo Hospitalario Universitario, A Coruña. The specimens included 49% of squamous cell carcinomas (SCC), 48% of adenocarcinomas and 3% of large cell carcinomas based on WHO criteria of the histopathological classification. The tumors were classified as stage I (55% of tumors), and stage II and III (45% of tumors) based on the American Joint Committee on Cancer guidelines for postsurgical, tumor-node-metastasis (TNM) [38].

### Immunohistological analysis of HLA-I, and PD-L1 expression in tumor tissue samples

We analyzed 68 NSCLC tumors in total. All 68 samples were analyzed for HLA expression (Table 1) and 52 samples for PD-L1 expression (Table 2). Most of the tumor specimens were cryopreserved and in some cases both frozen and paraffin-embedded samples were used for PD-L1 expression analysis. Tumor samples were taken from primary malignant lung tumors by excision of a fragment of tumor mass during the initial surgery. After thoracotomy and lung resection, half of the piece was immediately stored at –80°C. Frozen 4–8-μm-thick tissues sections were cut using a microtome-cryostat (Bright), allowed to dry at room temperature for 4–18 hours, fixed in acetone at 4°C for 10 min, and stored at –40°C. Immunohistological analysis was performed using the Biotin-Streptavidin System (supersensitive Multilink HRP/DAB kit, BioGenex, The Hague, The Netherlands).

The following mouse monoclonal antibodies (mAbs) were used in cryopreserved tumor tissues to analyze HLA-I expression: W6/32 against HLA-A, B, and C heavy chain/B2M complex (a gift from Dr. Bodmer, Imperial Cancer Research Fund Laboratories, London, UK); GRH-1, which recognizes free and HLA class I heavy chain-associated b2-m chain, produced and characterized in our laboratory [39]; HC-10 against free heavy chain of HLA-B and C molecules (Nordic-MUbio, Rangeerweg, The Netherlands), anti-HLA-A which recognizes a subset of HLA-A locus-encoded gene products (1082C5) [40] and 42IB5 against HLA-B locus-encoded gene products 42-IB) [41]. Total loss of HLA-I molecules was defined by negative staining with W6/32 and GRH-1 mAbs according to the criteria established by the HLA and Cancer component of the 1996 International Histocompatibility Workshop [42]. PD-L1 expression was analyzed in 52 tumor samples. We used monoclonal anti-PD-L1 antibody (clone 22C3) and IHC kit PD-L1 22C3 pharmDx (DAKO) and EnVision FLEX System (DAKO, Santa Clara, USA ) on 2,5 μm sections following the manufacturer`s recommendations The sections were dried during 24 hours at 37°C followed by one hour of incubation at 60°C. Antigen retrieval was done using PT LINK reagent (DAKO) at low pH. The immunolabeled sections were digitally analyzed using Ultrafast Scanner 1.6 (Philips) and the images were visualized and photographed using the Intellisite Pathology solution Image Management System 2.4 (Philips). We classified tumors into 2 groups: PD-L1 negative tumors and tumors with positive or heterogeneous PD-L1 expression. Positive PD-L1 expression was defined as positive in tumor cells showing a membranous staining of any intensity.

### Study of tumor infiltration by immunohistochemistry

All 68 frozen tumor specimens were analyzed for the presence of tumor infiltrating cells using immunohistochemistry with a panel of specific antibodies. Positive labeling was evaluated by two independent observers who did not know the clinico-pathological characteristics of the corresponding patients. The observers evaluated tissue tumor infiltrates with 10x and 40× objectives by analyzing tissue immunolabeling with the following antibodies: anti-CD45 (clone GRT2 produced in our laboratory) [43], anti-CD45RO (Thermo Fisher, Waltham, Massachusetts, USA), anti-CD8 (clone C8/144B DAKO, Glostrup, Denmark), and anti-CD3 (clone F7.2.38, DAKO, Glostrup, Denmark). Whole tumor sample infiltration was evaluated in 5 different fields under 40x objective and scored as: + (≤30 cells/field), ++ (30–70 cells/field), +++ (70–120 cells/field) and ++++ (≤120 cells/field). We determined an infiltration score (1–4) for each marker per tumor sample by assigning one point to each cross (+) for subsequent statistical analysis. In addition, for we counted the number of CD8 positive T cells per microscopic field at a 400x magnification. We calculated the average value of the five representative fields examined for each slide.

### Baseline and IFN-gamma induced upregulation of HLA-I and PD-L1 in human lung cancer cell lines

The following human lung cancer cell lines were used in this study: A549 (adenocarcinoma), A427 (epidermoid carcinoma), CALU-1 (epidermoid carcinoma), CALU-6 (large cell carcinoma), SK-LU-1 (adenocarcinoma), and SK-MES (adenocarcinoma). All cell lines were obtained from the American Type Tissue Collection. Cells were maintained in Dulbecco’s Modified Eagle Medium (Sigma-Aldrich) or RPMI medium supplemented with 10% FBS (Life Technologies), 2 mmol/L glutamine (Sigma-Aldrich), and antibiotics. In some experiments, cell lines were treated with 500 U/mL IFN-γ for 48 hours (Sigma-Aldrich) before FACS analysis. PE-conjugated anti-CD274 (BD Bioscience, San José, California, USA) and APC-conjugated anti HLA-ABC monoclonal antibody, (purchased from e-Bioscience) were used for PD-L1 and HLA-I analysis, respectively (eBioscience, Sandiego, California, USA). Cells were incubated with primary antibodies for 30 min at 4°C and analyzed on a FACSCanto cytometer (BD Biosciences). Isotype-matched nonimmune mouse IgGs conjugated with FITC and APC served as controls. The results are expressed as mean fluorescence intensity (MFI).

### HLA-I genomic typing in human cancer cell lines

DNA from NSCLC cell lines were used to perform HLA-I genomic typing with the LIFECODES HLA-A, -B and -C Typing Kits–Rapid (IMMUCOR, Stanford, USA) following the manufacturer’s instructions. The Luminex 100/200™ System, based in xMAP Technology (Luminex^®^, Austin, Texas, USA) was used to analyze HLA-I typing, and consequently, to detect haplotype, locus or allele losses in HLA-I genes in tumor samples.

### Array-based comparative genomic hybridization (array-CGH)

DNA samples from lung cancer cell lines and controls were genotyped using the Illumina Infinium assay on the Immunochip according to manufacturer protocol, which detects about 200,000 SNPs selected based on GWAS of the diseases of the immune system. Data for loss of heterozygosity (LOH) analysis and copy number (CN) analysis were obtained from the Illumina Genome studio software as “theta” and “R” values. “Theta” represents the B-allele frequency and “R” the joined fluorescence intensity of both channels. While “theta” can be interpreted directly to detect LOH using e.g. BCFtools [PMID:26826718], “R” needs to be compared to a standard to detect regions of copy number loss or gain. We used immunochip data from 1632 unrelated samples of European ancestry to obtain a median fluorescence value per probe to create such a standard and to subsequently obtain Log-ratios. A Log-ratio distribution around zero can be regarded as CN neutral, while chromosomal intervals of mainly positive (or negative) log-ratios can be interpreted as CN gains (or loss). Chromosomal stretches of B-allele frequencies with values of mainly zero or one can be interpreted as LOH.

### Statistical analysis

All statistical analyses were performed using the Statistical Package for the IBM-SPSS Statistics Ver.21. To check for normality we used Shapiro Wilk and Kolmogorov-Smirnov tests. Variables with normal distribution are expressed as means with standard deviation. Variables with non-normal distribution are expressed as medians and interquartile range. In the case of quantitative variables, differences among all study groups were evaluated by using a *t*-test in case of normality or a non- parametric test (Wilcoxon or Mann Whitney Test) for non-normal variables, when two groups were compared. We performed Kruskal-Wallis test in case of non-normal variables when more than two groups were compared.

Categorical variables, such as sex, smoking history, tumor stage, PD-L1 expression were coded in two groups and analyzed using the chi-square (X^2^) or Fisher’s exact test in case when the validity criteria were not reached. Differences were considered statistically significant at *p* < 0.05.

## SUPPLEMENTARY MATERIALS FIGURE AND TABLES


