# Supplementary material for: HLA class I loss and PD-L1 expression in lung cancer: impact on T-cell infiltration and immune escape
Source: Oncotarget. 2017 Dec 19;9(3):4120–33. doi: 10.18632/oncotarget.23469 (PMC5790526; doi:10.18632/oncotarget.23469)
Supplement: Supplementary file 1 [file oncotarget-09-4120-s001.pdf]

HLA class I loss and PD-L1 expression in lung cancer: impact on T-cell infiltration and immune escape

SUPPLEMENTARY MATERIALS

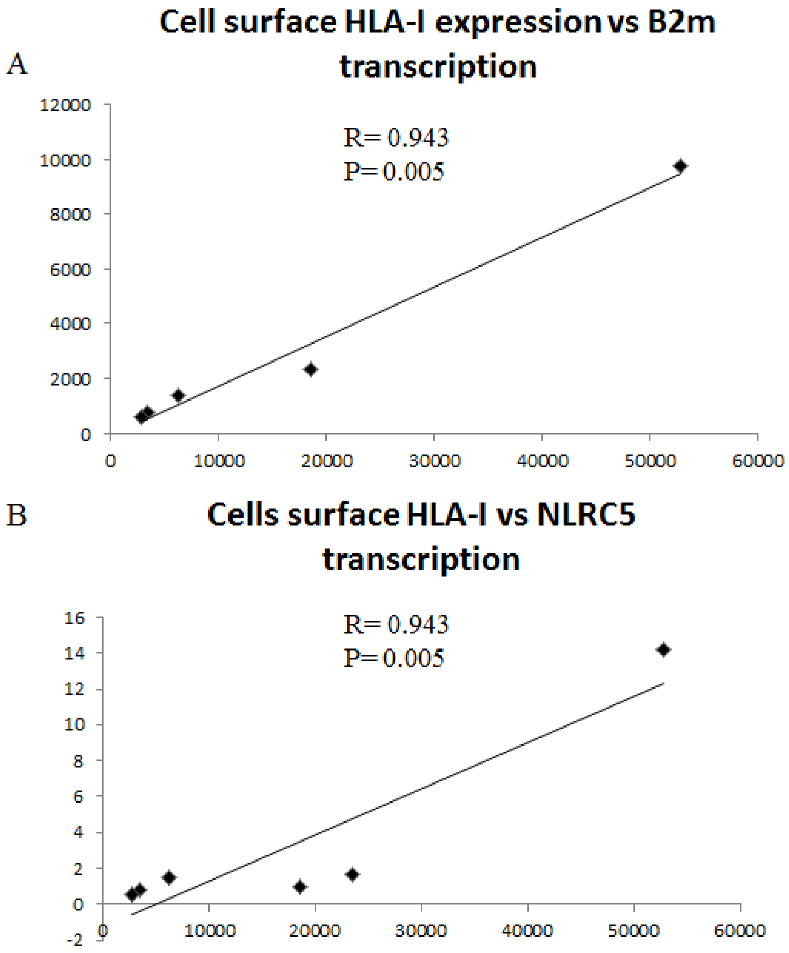

Supplementary Figure 1: Correlation between HLA surface expression and transcription levels of B2M and NLRC5. (**A**) Spearman correlation analysis between cell surface HLA-I expression and B2M transcription levels in lung cancer cell lines. (**B**) Spearman correlation analysis between cell surface HLA-I expression and NLRC5 transcription levels in lung cancer cell lines. HLA-I expression is shown as mean florescence intensity (MFI) and the levels of mRNA expression of the B2M and NLRC5 genes as Ct normalized against the housekeeping gene GUS used as a control.

Supplementary Table 1: Baseline and IFN-gamma induced cell surface HLA-I and PD-L1 expression in lung tumor cell lines (presented as Medium Fluorescence Intensity, MFI)

Cell line		Baseline	IFN-gamma
A-427	HLA	2801	10687
	PD-L1	257	271
SKMES	HLA	3421	19534
	PD-L1	256	378
A-549	HLA	6226	22585
	PD-L1	1197	1335
CALU-6	HLA	18568	41943
	PD-L1	1530	2702
SKLU-1	HLA	23412	26438
	PD-L1	474	356
CALU-1	HLA	52825	71168
	PD-L1	2394	6696

Supplemmentary Table 2: HLA genomic typing of lung cancer cell lines

Line cell	A1	A2	B1	B2	C1	C2	DRB1 1	DRB1 2	DQB1 1	DQB1 2
A-427	3:01	33:01:00	35:03:00	35:03:00	12:03	12:03	4:04	13:02	3:04	6:03
A-549	25:01:00	30:01:00	18:01	44:03:00	12:03	16:01	7:01	11:04	2:02	3:01
CALU-1	26:01:00	29:02:00	15:01	44:03:00	3:04	16:01	7:01	14:04	2:02	5:03
CALU-6	1:01	1:01	8:01	8:01	7:01	7:01	3:01	3:01	2:01	2:01
SKLU-1	24:02:00	24:02:00	40:02:00	40:02:00	2:02	2:02	13:01	13:01	6:03	6:03
SKMES	3:01	3:01	7:02	7:02	7:02	7:02	16:01	16:01	5:02	5:02
